# Directional organization and shape formation: new illusions and Helmholtz's Square

**DOI:** 10.3389/fnhum.2015.00092

**Published:** 2015-03-03

**Authors:** Baingio Pinna

**Affiliations:** Department of Humanities and Social Sciences, University of SassariSassari, Italy

**Keywords:** shape perception, Gestalt psychology, perceptual organization, visual illusions, directional organization

## Abstract

According to Helmholtz's Square illusion, a square appears wider when it is filled with vertical lines and higher when filled with horizontal lines (Helmholtz von, 1866). Recently, Pinna (2010a) demonstrated that the grouping of small squares on the basis of the similarity principle influences also perception of their shape and of the whole emerging shapes. The direction imparted by grouping is the main attribute that influences the shape by polarizing it in the same direction both globally and locally. The rectangle illusion is opposite to what expected on the basis of Helmholtz's Square illusion. Aim of this work is to solve the antinomy between the two sets of illusions and to demonstrate a common explanation based on the interaction between different sources of directional organization. This was accomplished by introducing some new phenomena and through phenomenological experiments proving the role played by the directional shape organization in shape formation. According to our results, Helmholtz's square illusion shows at least two synergistic sources of directional organization: the direction of the grouping of the lines due to their similarity of the luminance contrast and the direction of the grouping of the lines due to the good continuation.

## The rectangle illusion and Helmholtz's Square antinomy

Gestalt psychologists first investigated and developed a theory of object perception and perceptual organization. They approached the problem of visual objects in terms of figure-ground segregation and grouping. Rubin (1921) studied the object formation by investigating what appears as a figure and what as a background. He discovered the so-called figure-ground principles: surroundedness, size, orientation, contrast, symmetry, convexity, and parallelism.

**Figure 1:**
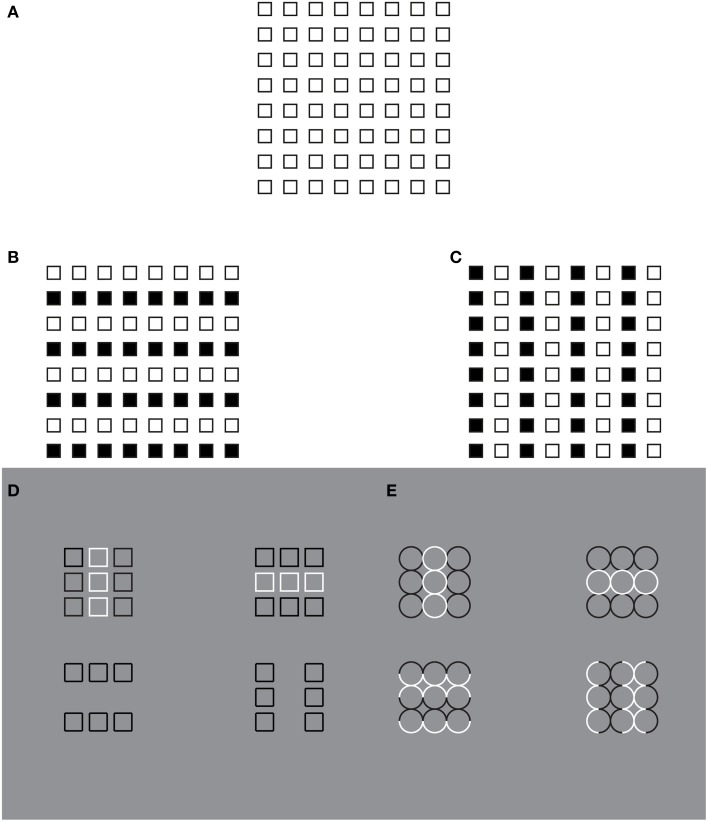
**The grouping direction “deforms” both the small and the large squares by widening/lengthening their base and creating horizontal/vertical rectangles**. This phenomenon was called “**rectangle illusion**.” Similar effects can be perceived in the circles. For further details please see the text.

In Figure 1A, small squares are unanimously perceived. Most of the previous principles contribute to their emergence as solid figures segregated from the white and empty background. In addition, Figure 1A shows a further kind of visual organization, considered crucial by Gestalt psychologists: the grouping of the elements in a square (Wertheimer, 1923). In this figure, the square array is seen as a whole and, at the same time, as a collection of elements spatially arranged in a precise regular order. In combination Figure 1A is perceived as a single object made up of many elements, i.e., the parts of the whole. The large square is thus the overall and holistic object popping out from the grouping of the squares that become its elements or components. Therefore, many elements appear as one. This is the uniqueness of a multiplicity that depends on the grouping.

The main questions related to the dynamics of grouping are the following: how do the elements in the visual field “go together” to form an integrated percept? How do individual elements create larger wholes? Wertheimer, in accordance with the Gestalt approach, answered these questions in terms of general principles: proximity, similarity, good continuation, closure, symmetry, convexity, Prägnanz, past experience, exhaustiveness, common fate, and parallelism. All these principles contribute with different strength to the whole formation of Figure 1A.

The holistic, symmetrical and balanced grouping of Figure 1A do not reveal any preferential direction among the squares, although row or, alternately, column organizations (mostly elicited by the proximity principle), rather than the diagonal one, can be induced through the visual attention. Every kind of possible organization in the vertical or horizontal direction can be easily changed when the attention is switched to one or to another possible direction. This entails that under homogeneous conditions, no preferred directional (horizontal vs. vertical) organization emerges, according to the fact that all the principles are perfectly counterbalanced along these directions. However, this is not true for the oblique organization that is weaker than the vertical/horizontal one, due to the proximity principle, according to which only closest elements such as the horizontal/vertical ones tend to group together.

By introducing a discontinuity within the homogenous pattern of Figure 1A (see Figures 1B,C), the grouping changes. Now, two large squared shapes made up of rows (b) or columns (c) of small squares are clearly perceived on the base of the similarity of the elements (filled vs. empty or black vs. white squares). As a matter of fact, the Gestalt principle of similarity (Wertheimer, 1923) states that, all else being equal, the most similar elements (in color, brightness, size, empty/filled, shapes, etc.) are grouped together.

On a closer observation of Figures 1B,C, the shapes of the small and large (whole) squares were reported spontaneously as not isotropic (directional invariant) but with a clear directional organization (see also Pinna, 2010a,b), according to which the inner organization in rows or columns appears not only oriented but also elongated in the same direction as the one of the perceptual grouping. In other words, the grouping in rows “deforms” both the small and the large squares by widening their base and creating horizontal rectangles. On the contrary, the grouping in columns induces a perceptual lengthening of the height of both small and large squares creating vertical rectangles. This phenomenon was called “rectangle illusion” (Pinna, 2010a, 2012). Indeed, these results emerge more saliently through a comparison of Figures 1B,C with the control of Figure 1A and are even stronger by focusing the attention on the small array of 3 × 3 squares on the left or right upper side of Figures 1B,C. The rectangle illusion is definitively corroborated through the new conditions illustrated in Figures 1D,E.

This illusion suggests that the grouping by similarity can influence the perceived shape by making squares to appear as rectangles. Therefore, it can be concluded that grouping and shape are strongly related to one another, though they have been considered as placed at different levels of visual organization. As a matter fact, the grouping considered in the meaning of Gestalt psychologists cannot make any prediction about the shape of the rectangle illusion. Briefly, the role of the Gestalt principles was to define the rules of “what is it, or what stays with what” i.e., the grouping and not the shape. On the other hand, the notion of “whole” due to grouping was considered phenomenally different from the one of the shape. These differences might be attributed to the choice of the term “grouping” that is regarded as synonym of “aggregation” and “assembling.” More in details, while the grouping puts together elements that become “parts” within a holistic percept, the shape emerges as a result of a global perceptual process imparting to the whole a unitary form along the boundary contours. This was not a literal or a mere linguistic or philosophical distinction but a scientific distinction that has had crucial consequences in terms of experimental phenomenology, for example in relation to the following conditions, and neural circuitry.

**Figure 2:**
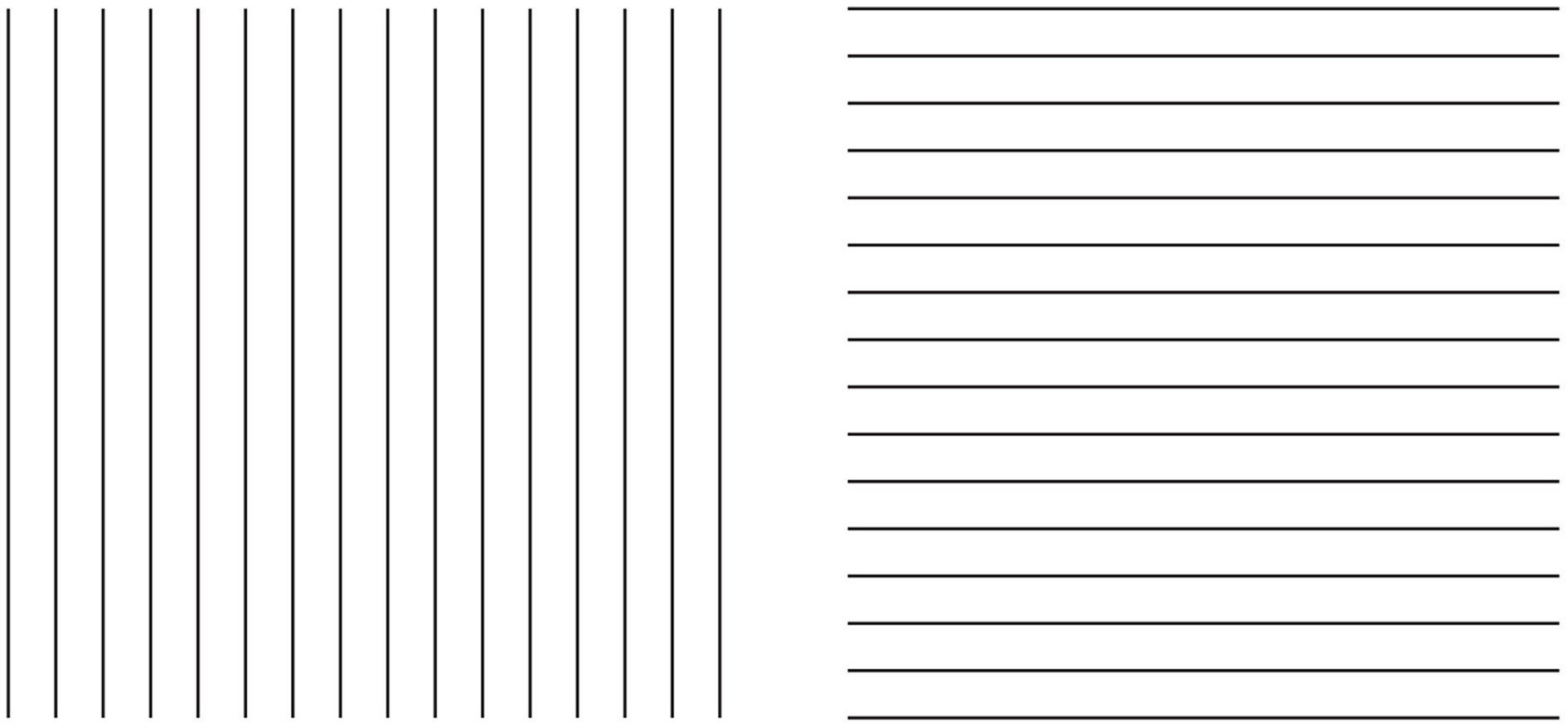
**Helmholtz's square illusion shows the widening of the square made up of vertical lines and its lengthening when the lines are horizontal**.

The rectangle illusion demonstrated in Figure 1 can be considered as related to Helmholtz's Square (Figure 2), according to which a square appears wider when it is filled with vertical lines and higher when filled with horizontal lines (Oppel, 1854–1855; Helmholtz von, 1866; Kristof, 1961; Da Pos and Zambianchi, 1996; Thompson and Mikellidou, 2011).

In spite of the apparent similarities, the rectangle illusion is totally different from Helmholtz's Square: (i) the whole shape distortion is induced by grouping and not by filled vs. unfilled space; (ii) the direction of the illusory distortion is the opposite of the one perceived in Helmholtz's Square, and (iii) the shape distortion involves both the small squares and the whole square shape.

By comparing the rectangle illusion and Helmholtz's Square an unsolvable antinomy seems to emerge. If a square composed of horizontal lines (rows) appears taller and narrower than an identical square made up of vertical lines (columns), why does a square made up of small squares arranged in rows appears, on the contrary, wider than an identical pattern when the elements are vertically arranged in columns? This question is appropriate if the grouping is considered not only as a synonym of “aggregation” and “assembling,” but also as a shape formation that, under these conditions, appears made up of rows and columns. Therefore, if the two effects are considered in terms of directional organization, then the explanation of one effect (i.e., the rectangle illusion) leaves the other unexplained and *vice versa*. By explaining one phenomenon, the other becomes a counterexample. The same problem occurs when other kinds of explanations are considered.

The main purposes of this work are (i) to solve the antinomy between the two sets of illusions, (ii) to demonstrate a common explanation based on a deeper understanding of the directional organization and (iii) to prove its more general role in shape formation. Finally, this will be accomplished through phenomenological experiments and by introducing some new phenomena.

## General methods

### Subjects

Different groups of 14 naive subjects each ranging from 20 to 27 years of age participated in each experiment described in the next sections. Subjects were about 50% male and 50% female and all had normal or corrected to normal vision. All participants signed an informed consent.

### Stimuli

The stimuli were the figures shown in the next sections. The stroke width was ~6 arcmin. The luminance of the white background was ~122.3 cd/m2. Black contours had a luminance value of ~2.6 cd/m2). The stimuli were displayed on a 33 cm color CRT monitor (Sony GDM-F520 1600 × 1200 pixels, refresh rate 100 Hz), driven by a MacBook computer with an NVIDIA GeForce 8600 M GT, in an ambient illuminated by an Osram Daylight fluorescent light (250 lux, 5600°K). They were viewed binocularly and in the frontoparallel plane at a distance of 50 cm from the monitor. In the adjustment/matching experiment, described below, the size of the matching empty square was chosen randomly between 25 and 30 mm.

### Procedure

#### Phenomenological task

The subjects' task was to report spontaneously what they perceived for each stimulus by giving, as much as possible, an exhaustive description and, if necessary, to answer the questions asked by the experimenter. Subjects were also instructed to scale the relative strength and salience (in percent, where 100 is the maximal salience and 0 the minimal) of the perceived alternatives, if there were any, and the relative confidence and appropriateness of the responses were also taken into consideration.

#### Scaling task

Subjects were also instructed to scale the relative strength or salience of the perceived illusion in percent. The reference values were: 0 to represent the absence of the illusion; 100 to represent the maximal strength of the illusory appearance.

#### Adjustment/matching task

The conditions illustrated in **Figure 14**, crucial for our purposes, were compared and measured through an adjustment/matching experiment. Subjects' task was to adjust the conditions shown in **Figure 14** (target figures) presented side by side (left/right and up/down positions) to a match figure (the reference square) so that they looked of the same vertical/horizontal size of the match figure. Each subject matched 4 positions (left/right and up/down) × 6 stimuli in random order. The initial stimulus and position were randomly chosen. Each subject was tested for 20 trials. To measure the perceived effects, on each trial, the observers adjusted the length of the base and the height of the reference square to match the whole shape of the bunch of vertical/horizontal lines of each stimulus, using the left/right up/down arrow keys on a computer keyboard. For example, each time the observer pressed the up arrow key, the height of the match square got longer by 1 pixel. In a similar manner, the down arrow key made the height shorter. The horizontal size of the match square changed accordingly each time the subject pressed the left/right arrow key. By pressing the spacebar key, the subject confirmed the adjustment. The next random trial of one of the six target stimuli started immediately after. No time limit was given for the adjustments.

In the next sections, the descriptions/results are included within the main text to aid the reader in the stream of argumentations. The edited descriptions were judged to provide a fair representation of those provided by the observers. If not specified, the descriptions reported in the next sections were those spontaneously communicated by 12 out of 14 subjects and judged highly appropriate (more than 90%).

During the phenomenological and scaling experiments subjects were allowed: to make free comparisons, confrontations, afterthoughts, to see in different ways, to match one stimulus with others, to make variations and comparisons in the observation distance, etc. The subjects could also receive suggestions/questions from the experimenter. All the variations and possible comparisons occurring during the free exploration were noted down.

Subjects were tested individually. No time limit was set to the descriptions and the scaling, which occurred spontaneously and fast. The stimuli were shown continuously during the description task. Details and variations among experiments related to the subjects, the stimuli and the procedure will be reported more in details in the next sections together with the results of each experiment and a theoretical discussion.

## Directional organization and shape formation

In this section, the strong binding between grouping and shape perception will be studied through some recent and new phenomena. According to our hypotheses grouping can influence shape and, *vice versa*, through the directional organization that can be considered as a basic principle of shape formation.

**Figure 3:**
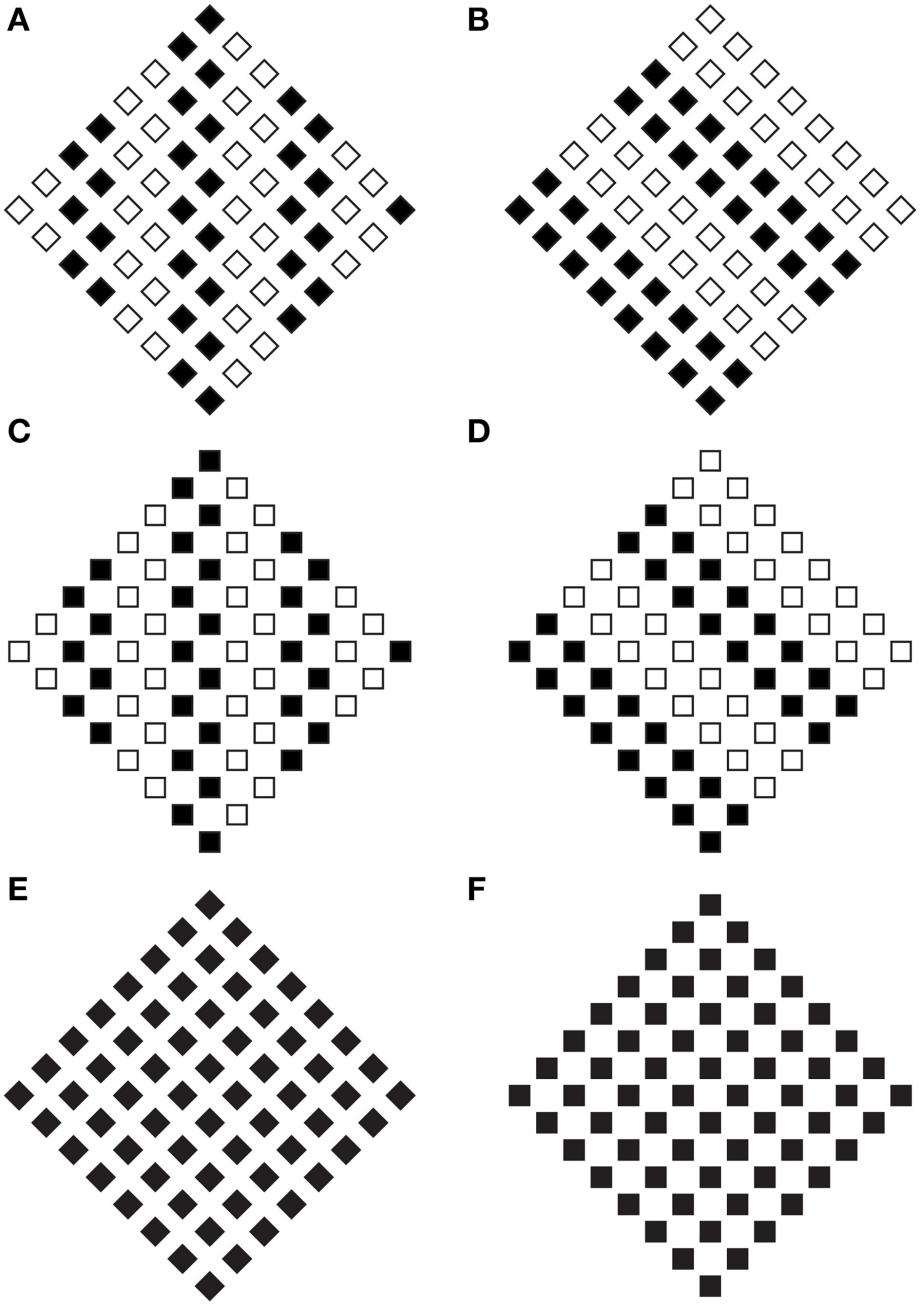
**The grouping by similarity induces the directional organization that induces diamonds and rotated squares in the same geometrical patterns**. For further details please see the text.

The rectangle illusion, shown in Section The Rectangle Illusion and Helmholtz's Square Antinomy, is not only a simple deformation effect, but mostly an instance of all the effects induced by the directional organization that, all else being equal, we assume as one of the main sources of shape formation. This is demonstrated in Figure 3, where the grouping by similarity induces directional organizations along the diagonals or along the sides of the check elements highlighting different part components of each check, respectively sides and angles. In Figure 3A, geometrically, square shapes (45° rotated), i.e., with four sides having the same length, are arranged in a large diamond that is again a 45° rotated square shape. Perceptually, the vertical organization along the diagonals/angles of both the single and whole components, on the base of the similarity principle, strengthens the perception of diamonds both in each single checks and in the whole arrangement of checks. These results emerge more clearly by comparing this figure with the control illustrated in Figure 3E. Moreover, similarly to the rectangle illusion, the vertical directional organization induces also a vertical deformation/elongation of the elements that are perceived not as 45° rotated squares but as rhombuses with different horizontal and vertical diagonals.

In Figure 3B, the same geometrical pattern of Figure 3A, but with an oblique directional organization, i.e., along the sides of the checks, due to the similarity principle, induces different percepts. Both the single checks and the whole arrangement are now perceived as squares rotated by 45°. Moreover, while in Figure 3A, the pointedness attribute of both the single and whole components stands out immediately, in Figure 3B, the sidedness and flatness of the same components emerge.

The phenomenal differences between a diamond and a square rotated by 45° are considerable. In a diamond the phenomenal emphasis is placed on the vertices showing its “pointedness,” whereas in the rotated square the emphasis is placed on the sides showing its “sidedness” (see also Pinna and Sirigu, 2011; Pinna, 2012). Therefore, a diamond and a square rotated by 45° are phenomenally two different figures because they show opposite properties and, thus, they need two different names.

These results clearly suggest that the directional organization accentuates and polarizes different shape attributes (sides or angles), thus inducing the kind of perceived shape. Similar effects occur in Figures 3C,D, where the diamond arrangements of squares (in Figures 3A,B there were diamond arrangements of diamonds) in the two kinds of grouping, along the sides (Figure 3C) and along the diagonals (Figure 3D), are perceived respectively as squares arranged in a large diamond and diamonds (or lozenges) arranged in a large square rotated by 45°. These results emerge more clearly through the comparison of these two figures with the control of Figure 3F.

There are other four possible systematic arrangements, i.e., square arrangements of squares and square arrangements of diamonds with two different similarity groupings. Under these conditions (not reported), the results support the predictions based on the directional organization induced by the principle of similarity.

These preliminary outcomes suggest that the directional organization could be considered like a meta-principle of shape formation, i.e., a principle of shape formation based on another principle of shape formation that is, under these conditions, the similarity. Therefore, one principle is nested within the other or, accordingly, one principle occurs when also the other occurs. There is another way to explain these results. The similarity principle, not only does it put together the more similar elements but also polarizes a direction and highlights or accentuates different components that elicit one or another shape attribute: sidedness and pointedness.

It is worthwhile to note that the previous results appears immediately related to the configural orientation effect (Attneave, 1968; Palmer, 1980, 1985, 1989; Palmer and Bucher, 1981), according to which the perception of the local spatial orientation is influenced by the global spatial orientational structure. Despite these similarities, there are at least two basic differences: in Figure 3 the spatial orientational structure among elements is kept constant, while in Figures 3A,B and in Figures 3C,D, only the grouping is varied. Moreover, the effects perceived in our stimuli not only do they involve the local spatial orientation (in the classical experiments on the configural orientation, the dependent variable is the direction of pointing of equilateral triangles) but also they enhance the shape perception of both the whole object and each single inner component. Consequently, it is more plausible that the configural orientation effect is considered as a subset of the directional organization. This suggestion is demonstrated through the next figures.

**Figure 4:**
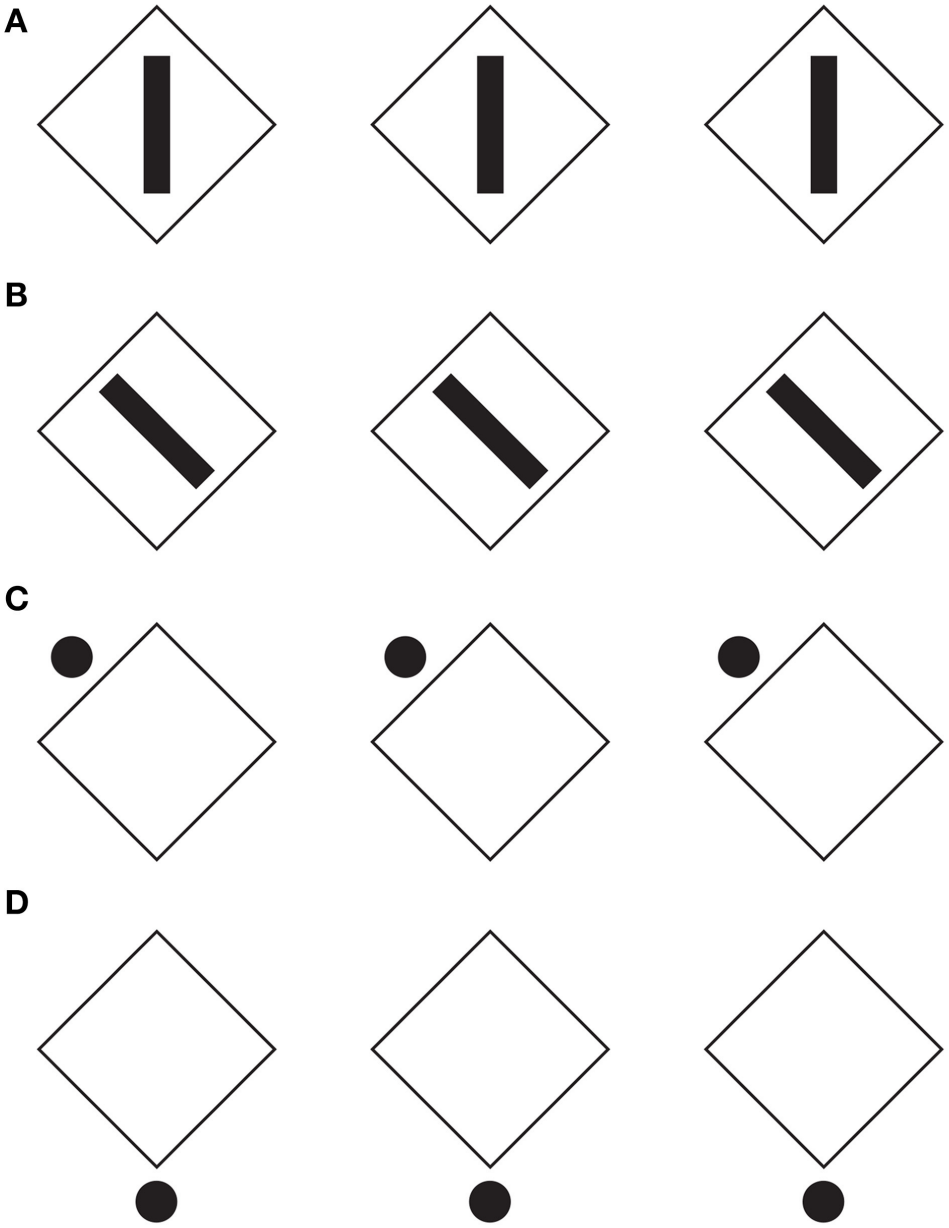
**The local directional shape organization creates diamonds (A,D) and rotated squares (B,C)**. For further details please see the text.

In Figure 4, the configural orientation effect is made ineffective by the local directional organization of each component. Therefore, the diamonds perceived in Figures 4A,D become rotated squares in Figures 4B,C, although all the shapes are geometrical equal.

**Figure 5:**
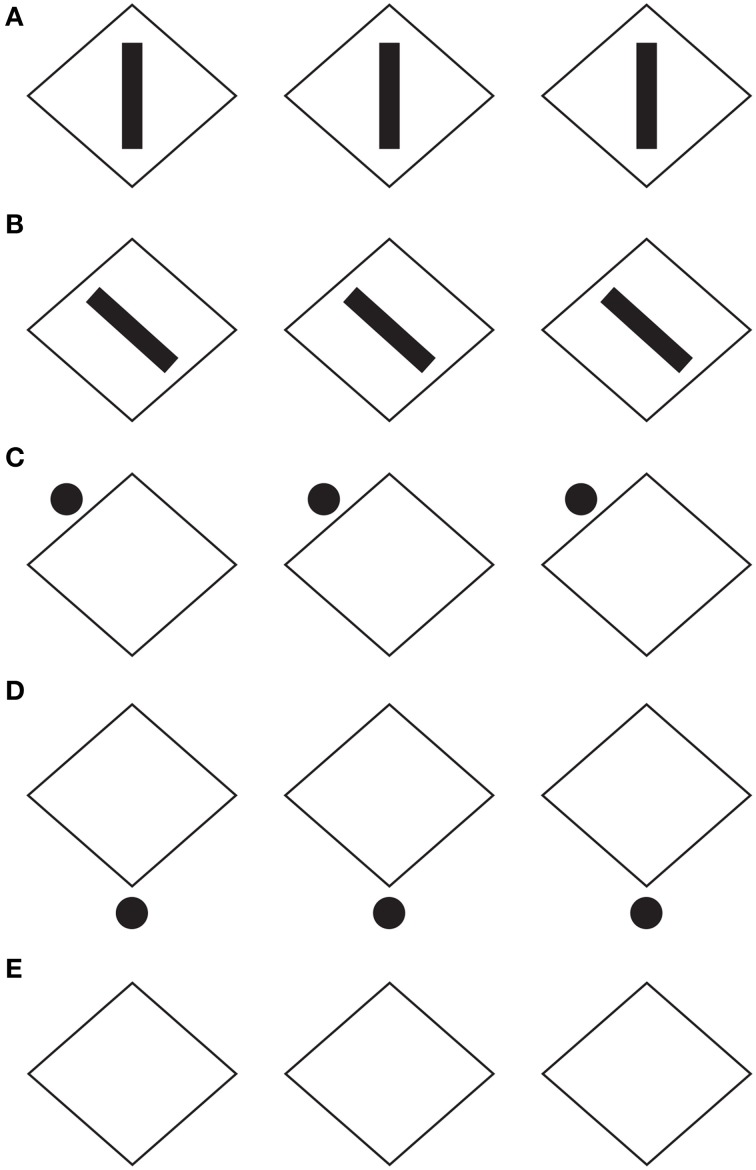
**The directional organization due to the filled elements induces diamonds and rotated squares as in Figure 4, although each shape is clearly perceived as a rhombus**. For further details please see the text.

Similar effects were also perceived in Figure 5, where each geometrical check of Figure 4 (i.e., the rotated square) was replaced with a rhombus (diamond or lozenge) with the longer diagonal placed along (synergistic to) the direction of the configural orientation, namely horizontally. In addition, the directional organization induces a regularity on the rhombuses by apparently elongating the shorter diagonal (Figures 5A,D; Figure 5E is the control). This effect, behaving similarly to the rectangle illusion of Figure 1, counterbalances the geometrical longer diagonal.

**Figure 6:**
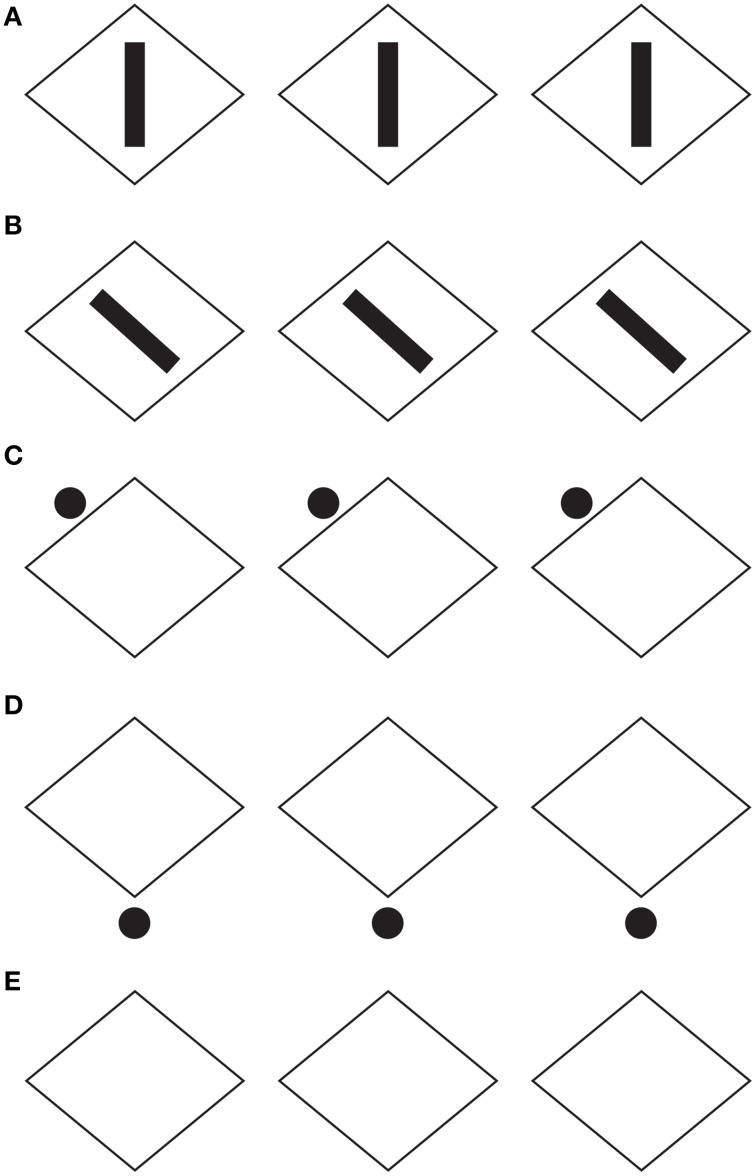
**The same results of Figures 4, 5 are also perceived when the length of the horizontal diagonal is further increased**. A set of skewed parallelograms are also shown. For further details please see the text.

When the length of the horizontal diagonal is further increased, as illustrated in Figure 6, and when the rhombuses appear as rotated square-like shapes (Figures 6B,C), a further effect was described: a set of skewed parallelograms. This result implies that the apparent shape of the checks depends on the three involved directional vectors, the one of the rotated square-like shape and the two of the diagonals of the rhombuses. Among them, the one induced by the black small rectangles and circles plays the main role.

There is a fourth vector synergistic with the horizontal diagonal, namely the one emerging from the configural orientation. However, on the base of the phenomenal results, the configural orientation can be considered as a secondary effect compared with the much stronger directional organization induced locally by the black rectangle and circles. Furthermore, while the configural orientation is mainly due to the larger reference frame of the whole organization of components, the directional organization induced from rectangles and dots/circles is due to the local directional organization related to each single elements. As a consequence, the whole effect can be considered as mostly due to the summation of these single local directional vectors.

**Figure 7:**
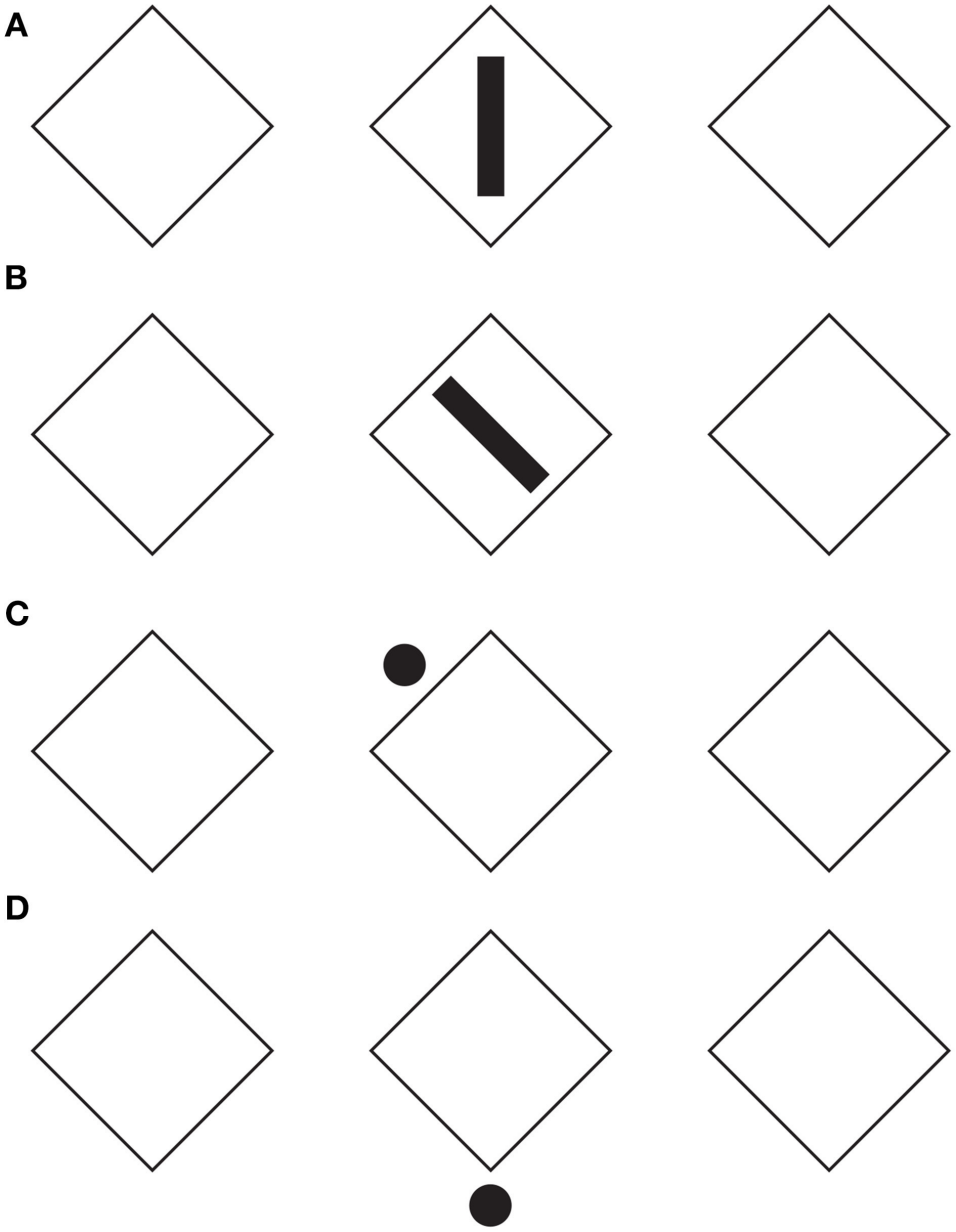
**The directional organization influences nearby figures**. For further details please see the text.

The role of the configural orientation effect is also very weak in Figure 7. Here, the same results of Figure 4 are perceived even if there is only one element either responsible for imparting the local directional organization and for inducing long-range effects and filling-in properties in all the elements of each row.

**Figure 8:**
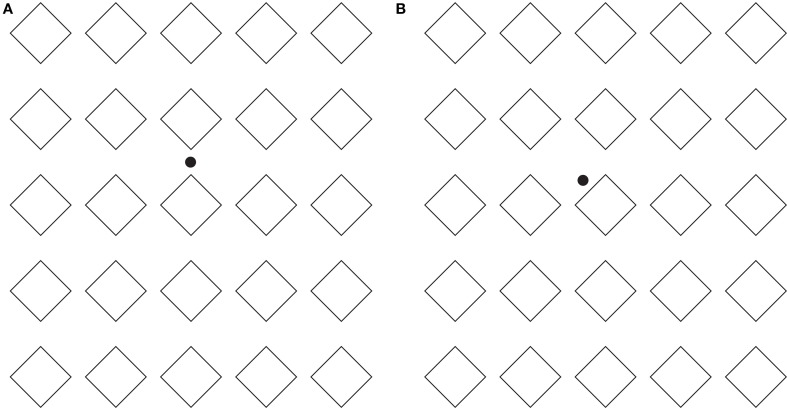
**The directional organization manifests long-range effects**. For further details please see the text.

This long range effect can be further extended as shown in Figures 8A,B, where respectively diamonds or rotated squares are perceived.

**Figure 9:**
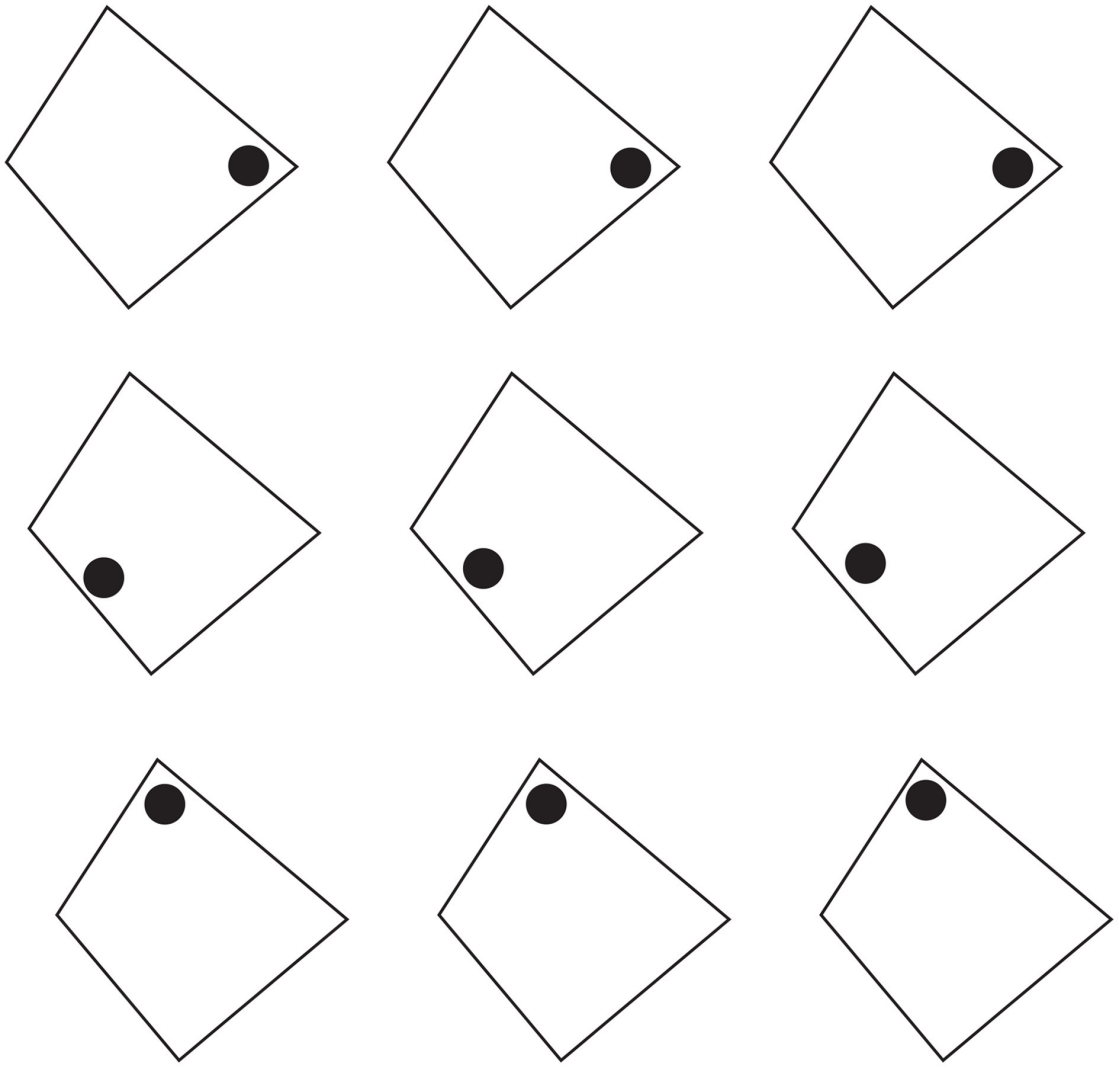
**The same figures, accentuated by black dots in different positions, are perceived as different irregular shapes**.

Other shape effects due to the directional organization are shown in Figure 9, where the same geometrical figures, accentuated by black dots in different positions of each shape, are perceived as rows of irregular shapes that appear different from one row to another (see also Pinna and Sirigu, 2011; Pinna, 2012).

The strength of the directional organization in accentuating different shape attributes can be seen in Movie 1, where the same 45° rotated square jumps stroboscopically clockwise around a virtual circle, while a small empty circle is placed alternately on one of its sides or on one of its vertices, thus accentuating different attributes of the diamond: sidedness and pointedness. Phenomenally, the perceived rotating shape switches alternately from a diamond to a rotated square. A similar results, can be perceived in Movie 2.

In the next section the notion of directional organization will be phenomenally deepened in relation to the grouping principles under different and more complex conditions.

## On the complexity of the directional organization

**Figure 10:**
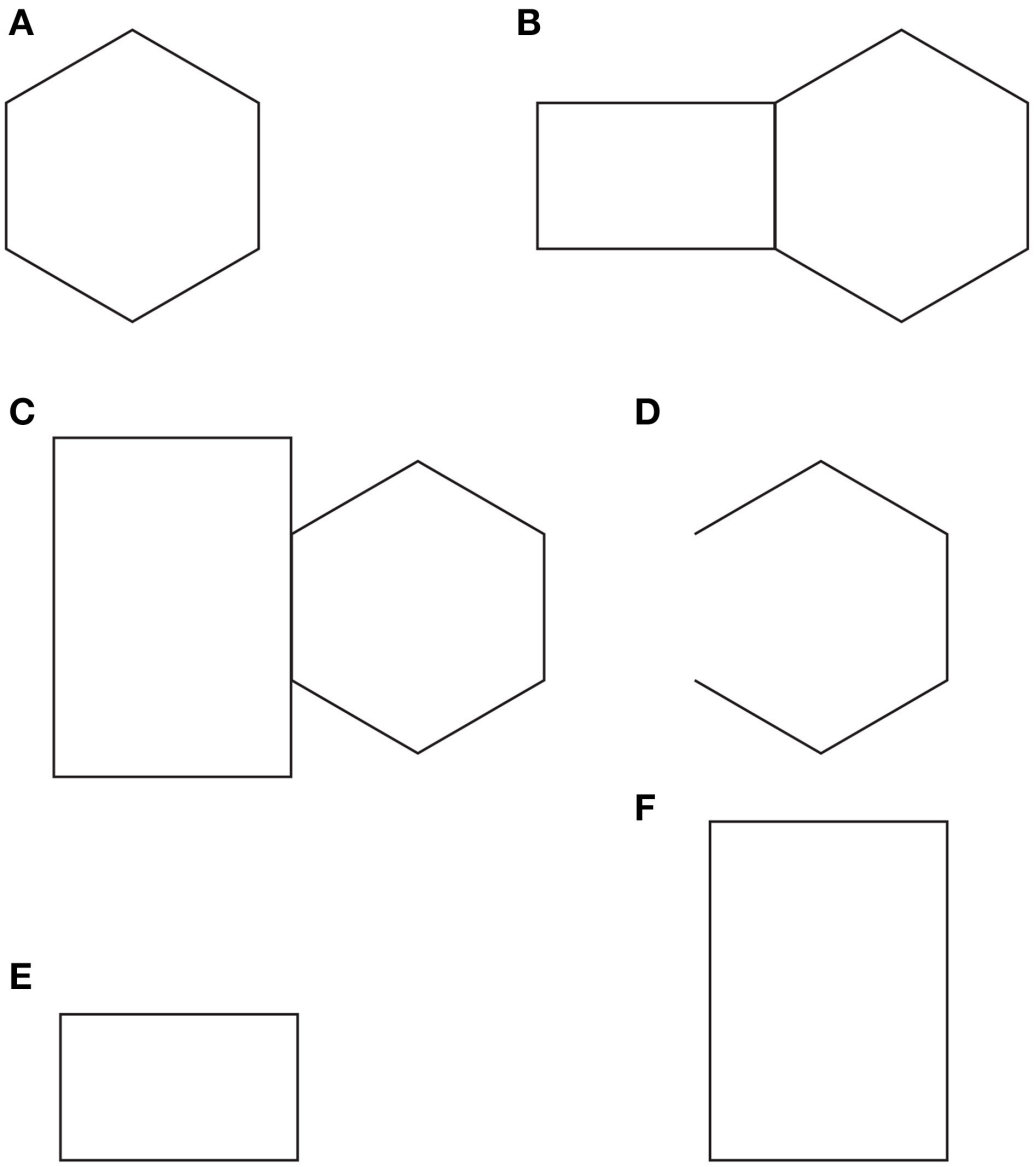
**Tessellation, occlusion and other sources of directional organization**. For further details please see the text.

In Figure 10A a regular hexagon is immediately perceive. In Figures 10B,C the same hexagon is shown juxtaposed to a rectangle under tessellation (Figure 10B) and occlusion (Figure 10C), where the intersecting contours belong (i) to both figures (the rectangle and the hexagon, Figure 10B), thus creating a communal ownership, or (ii) to one figure only (the rectangle in Figure 10C), therefore inducing the amodal continuation and completion of the hexagon. In Figures 10A,C, the hexagon appears respectively regular and symmetrical (Figure 10A), expanded and slightly elongated in the direction of the adjacent rectangle (Figure 10B), distorted, very elongated and asymmetrical in the direction of the occluding rectangle (Figure 10C).

These results and especially, the amodal completion condition of Figure 10C, demonstrates that the directional organization can be polarized not only by the rectangle placed on the left side of the hexagon and its orientation (Figure 10B), but also by the unilateral belongingness of the boundaries and, as a consequence, by the modal “absence” of the side of the hexagon that continues amodally behind the occluding rectangle (Pinna, 2013). Therefore, not only the grouping principles but also the unilateral ownership of the boundaries (Rubin, 1921; Nakayama and Shimojo, 1990; Spillmann and Ehrenstein, 2004) to the occluding rectangle induces a polarization of the occluded hexagon in the same direction of the missing side. This polarization is clearly perceived as a directional organization of the hexagon in Figure 10C, which changes its geometrical size and loses its shape symmetry and regularity. The directional organization is the basic principle of the expansion and shrinkage phenomena of the amodal completion under these conditions (Kanizsa, 1972, 1975, 1979, 1980; Pinna, 2013). However, though the amodal completion is the basic variable of the directional asymmetric effect of Figure 10C, it is not the only factor as demonstrated in Figure 10D, where a phenomenal outcome similar to the one shown in Figure 10C is perceived without amodal completion.

These results point out that the directional organization can be instilled by many different sources such as the spatial position of the rectangle with respect to the hexagon, the global orientation of the rectangle, the unilateral belongingness of the boundaries, the missing side and so on. All these sources can occur at the same time synergistically or one pitted against the other. Furthermore, they can be considered as oriented not only from the rectangle to the hexagon but also the other way round. More precisely, just as the spatial position and the shape of the rectangle influences directionally the shape and the size of the hexagon, so the same properties of the hexagon influence the rectangle. The two figures influence each other. In fact, by comparing the controls of Figures 10E,F with the rectangles of Figures 10B,C, the lengthening of the former (Figure 10B) and the widening of the latter (Figure 10C) can be clearly noticed.

A further comparison of Figure 10E with Figure 10F revealed that another source of directional organization contribute to determine the perceived size of the rectangles: their global orientation. On the basis of this further source of directional organization the higher rectangle of Figure 10F is also perceived as narrower.

The global orientation of the rectangle is also expected to affect the size (height and width) of the hexagons of Figures 10B,C. However, the orientation of the rectangles interacts with the source of the directional organization previously described, i.e., the unilateral belongingness of the boundaries and the amodal continuation of the missing side that induce a stronger directional effect. Nevertheless, the effects of the global orientation can also be visible. In fact, the hexagon of Figure 10B appears wider than the hexagon of Figure 10A, and the one of Figure 10C is perceived slightly higher than the one of Figure 10B. This last effect is strongly weakened by the amodal source of directional organization.

## Antinomies deduced from the directional organization: toward a solution

The notion of multiplicity of sources of directional organization and, to be more specific, the previous remarks about the global orientation of the rectangles bring the discussion back to the antinomic effects, already mentioned in section 1, between Figure 1 and the Helmholtz's Square of Figure 2, i.e., between the elongation of both the small and large shape squares in the same direction of the perceptual grouping (cf., Figure 1) and the widening of the square made up of vertical lines and its lengthening when the lines are horizontal (Figure 2).

At this stage the question is: can the two opposite conditions be predicted and explained by the directional organization? Apparently, the answer is “no.” In fact, at a first sight the resulting effect of vertical/horizontal grouping of Figure 1 seems to be analogous to the vertical/horizontal contours in Figure 2, although in the first case, this is due to the similarity principle, while, in the second, to the good continuation. Therefore, if the grouping results are the same, then also the whole resulting shapes should be the same. Why are they opposite, then?

A more accurate phenomenological analysis of the two conditions reveals that while in Figure 1 there is only one source of directional organization, due to grouping by similarity, in Figure 2, there are instead two opposite sources. One source is local and it belongs to each line and to the good continuation of its direction, whereas the second is global and it is related to the direction of the juxtaposition of lines. If the local directional organization is horizontal, the global one is vertical and *vice versa*. In particular, the two kinds of directional organization can be phenomenally distinguished as follows. The local source is the direction of each line, while the global one corresponds to the vertical/horizontal arrangement and distribution of lines, whose orientation is opposite, i.e., horizontal/vertical. In other words, the global directional organization is the opposite of the local one and it defines the whole shape of the square. This implies that, if the directional organization of the whole shape follows the same direction of the global organization due to the juxtaposed distribution of lines, the expected results are the same as those of Figure 1, where the directional organization of the whole square follows the directional organization of the grouping of the small squares.

If this is true the answer to the previous question (can the two opposite conditions be predicted and explained by the directional organization?) is positive. As a matter of fact, the square with vertical lines is perceived wider because of the horizontal distribution of lines, and, *vice versa*, the square with horizontal lines appears higher because of the vertical juxtaposition of lines. The distribution of lines is in both cases the means of information, because only across these distributions there is information about the shape of the whole object. This also explains why the global organization of lines is much stronger than the local direction of each line.

**Figure 11:**
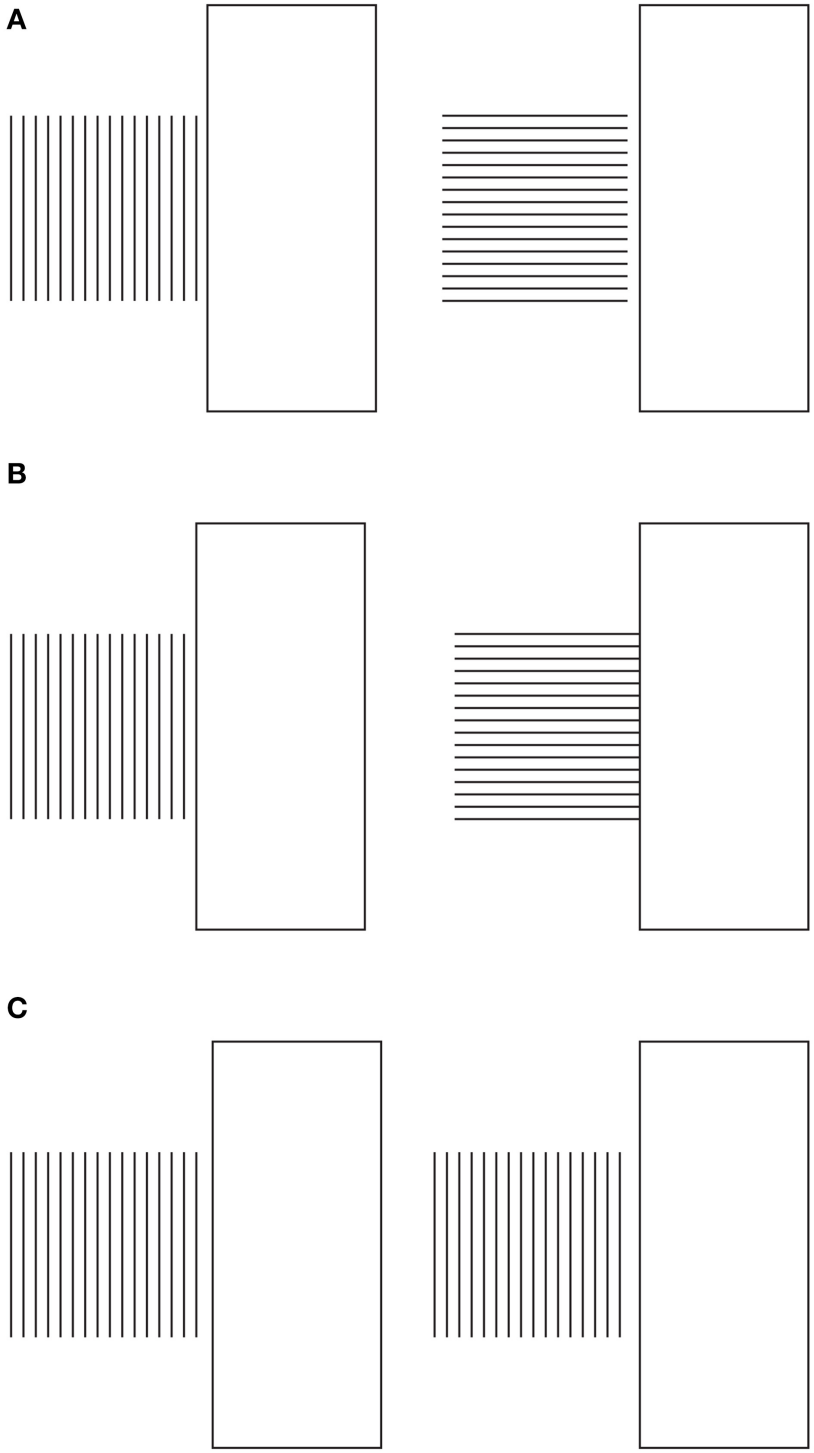
**The strength of the global directional organization can be perceived by comparing the strength of the amodal continuation of the vertical and horizontal arrangement of lines when a rectangle is placed nearby (for details see the text)**. For further details please see the text.

The conditions, illustrated in Figure 11A, clarify the meaning and the phenomenological strength of the two kinds of directional organization of Figure 2. The strength of the global directional organization can be perceived by comparing the strength of the amodal continuation of the vertical and horizontal arrangement of lines when a rectangle is placed nearby (Figure 11A). Under these conditions, the vertical bunch of lines clearly appears to continue amodally behind the large rectangle, while the horizontal lines do not show any continuation but are perceived as stopped in their geometrical terminations.

In Figure 11B, the two bunches of lines are placed juxtaposed to the rectangle, so as to induce more strongly their amodal completion behind the rectangle. Again the horizontal directional organization of the vertical lines shows that the amodal continuation behind the rectangle is much stronger than the one of the vertical directional organization. It was also noticed that Helmholtz's Square illusion persists or it is even stronger under this conditions. This is expected on the base of the argument we are following.

In Figure 11C, this strength is corroborated by the fact that the amodal continuation of the vertical lines is also perceived when the lines are clearly separated and, thus, disconnected by the rectangle. In spite of this separation, their directional organization shows a clear tendency to continue amodally and a sense of dynamic direction and polarization. This is also due to the fact that, although the rectangle is detached, it is placed along the same direction. This is the reason why, the resulting effect is stronger than the one illustrated in Figure 11A with the bunch of lines oriented vertically.

Furthermore, there is another effect supporting our arguments. By comparing the whole size and the perceived direction of the bunch of horizontal lines of Figure 11A with the bunch of vertical lines of Figures 11A,B, the former continues to appear higher and taller than the latter ones, despite the amodal continuation of the horizontal lines behind the rectangle. As a result, under these specific conditions, the global direction of the juxtaposed lines is a source of directional organization stronger than the unilateral belongingness of the boundaries that induces the amodal continuation.

**Figure 12:**
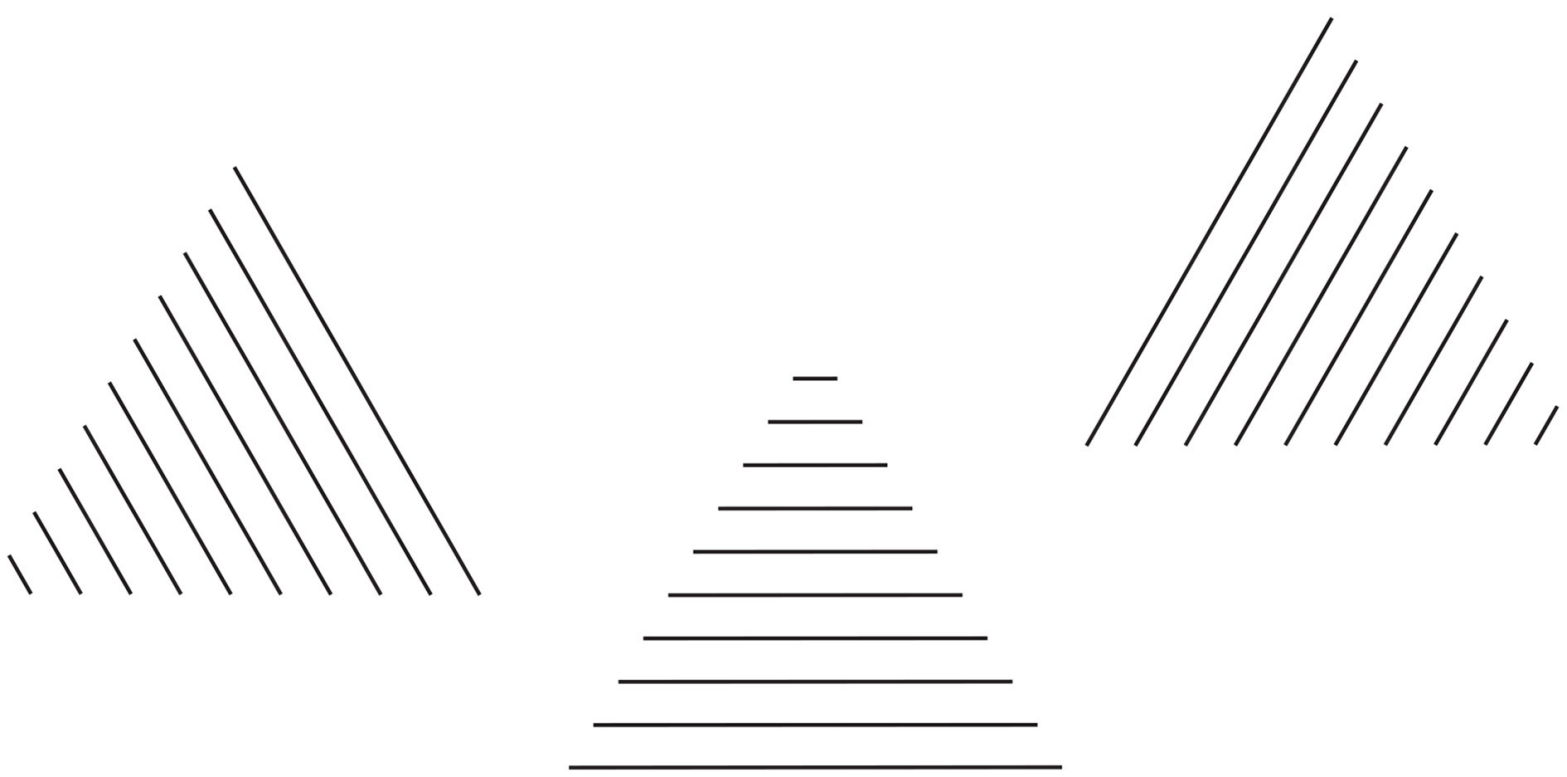
**The same equilateral triangle, made up of straight lines with different orientations, is perceived pointing in different directions on the basis of the direction of the arrangement of lines**.

A further demonstration of these sources of directional organization is shown in Figure 12, where the same equilateral triangle is made up of straight lines with different orientations. In this figure, all the triangles are oriented along the main directions of space, horizontal and vertical. The orientation of the triangles is not synergistic with the second source of directional organization that is the local orientation of the lines, which is parallel to one side at a time. The third source of directional organization, involving the whole shape is the directional juxtaposition of lines that is orthogonal to the direction of each line.

The main directions of space, horizontal and vertical can be considered as another important source of directional organization of the perceived shapes, as demonstrated by the square-diamond illusion (Schumann, 1900; Mach, 1914/1959; Palmer, 1999; Pinna, 2012), where the main space directions accentuate the sides when the square is perceived and the diagonals/vertices when the diamond is seen.

Among the three sources of directional organization the strongest is the third one, the directional juxtaposition of lines. It affects the global orientation of each triangle and, more particularly, the pointing of the equilateral triangles, which is one of the main attributes of shape perception, as demonstrated by the literature related to the configural orientation effect (Attneave, 1968). Nonetheless, the configural orientation effect cannot explain the pointing effects of Figure 12. Indeed, the pointing follows the directional juxtaposition of lines rather than the local orientation of lines.

**Figure 13:**
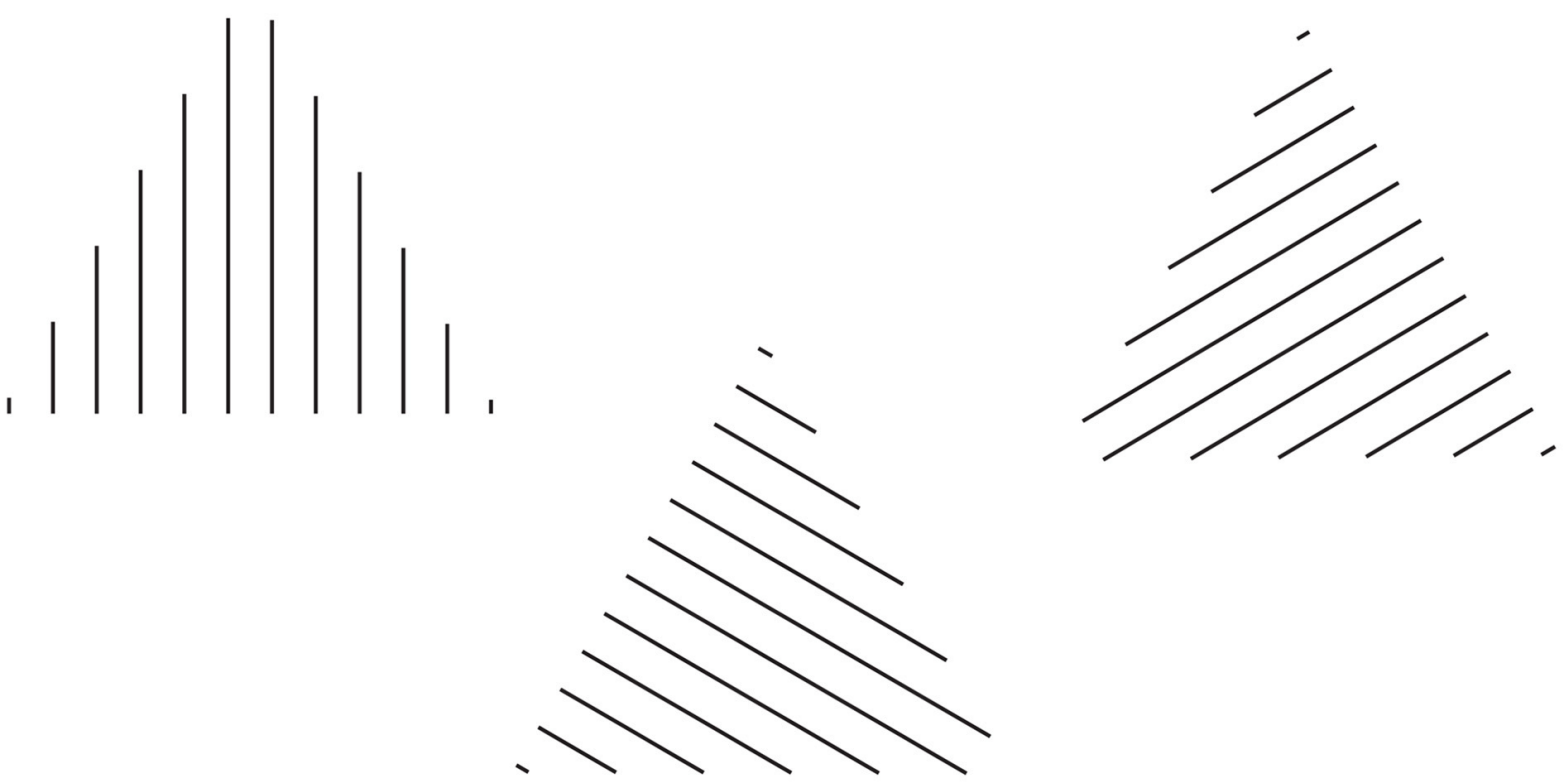
**The equilateral triangles are perceived pointing in different directions on the basis of the orientation of the lines**.

The source of the directional juxtaposition of lines is not necessarily in contrast to the local orientation of lines. In fact, due to the fact that both of these sources cannot affect the pointing at the same time, they assume different roles: the former becomes the height of each triangle and the latter defines its base. This is the result of some kind of compromise of a further interesting type of overall organization aimed to solve contradictory sources of directional organization. The opposite kind of directional cooperation is illustrated in Figure 13, where the roles between the two sources of directional organization are now reversed.

This kind of cooperation between directions reinforces the perceptual meaning of the pointing as a shape attribute by showing that the directional organization is an oriented direction that behaves like a Euclidean vector considered in the same meaning used in physics (see Pinna and Sirigu, 2011). The starting point of the oriented direction is placed on the base, defined by the local direction of each line. The direction, going from the starting and ending points, depends, in Figure 12, on the global directional juxtaposition of the lines. The ending or converging point is at the opposite pole to the starting point of the base. A phenomenal demonstration of these vectorial properties emerged by asking the subjects to orient an arrow according to the perceived orientation of the triangle of Figures 11, 12.

**Figure 14:**
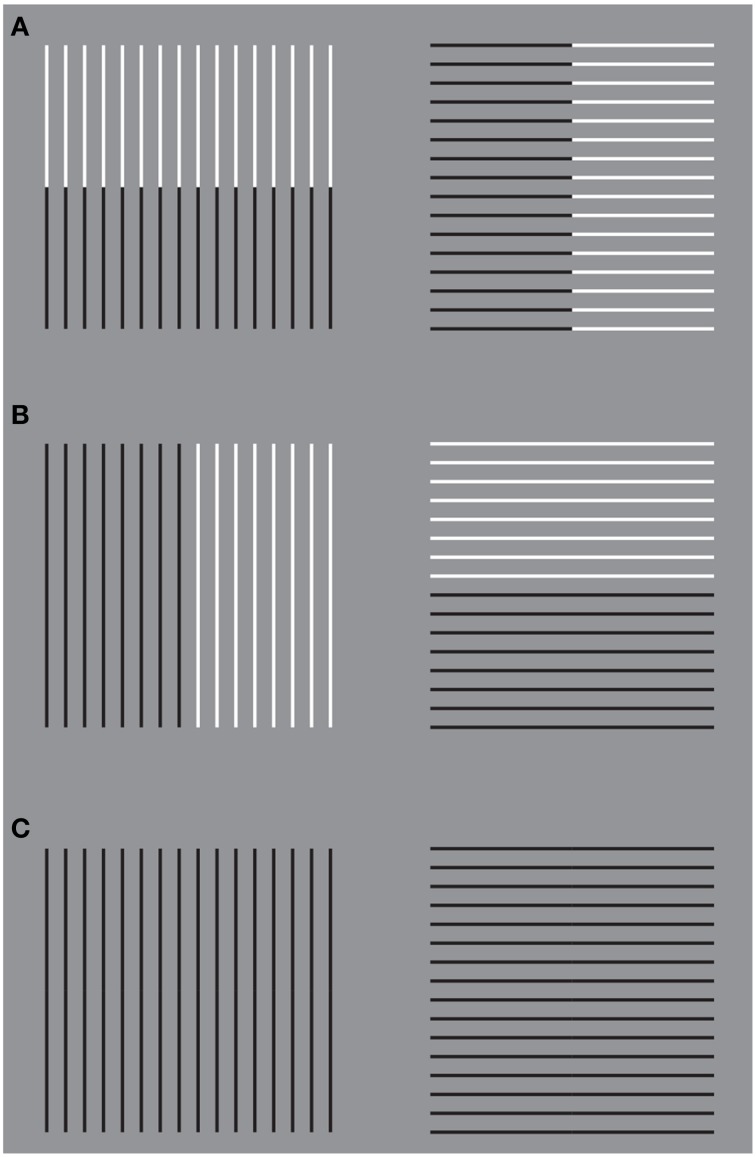
**The size effect of Helmholtz's squares can be strengthened (A) or weakened (B) in relation to the control (C) by introducing new sources of directional organization**.

A demonstration of another kind of interaction among different sources of directional organization in the form of subtraction and summation among different sources is illustrated in Figure 14. Under these conditions, Helmholtz's Squares show the following new sources of directional organization: the direction of the grouping of the lines due to their similarity of the luminance contrast and the direction of the grouping of the lines due to the good continuation. The grouping principles occur within the two other sources of directional organization previously studied: the local and global directions of the lines and of their juxtaposition. The two new sources are now pitted one in favor of the other as in Figure 14A or one against the other as in the case shown in Figure 14B. By comparing these critical conditions with the control shown in Figure 14C, a clear increasing (Figure 14A) or decreasing (weakening, almost annulling) as in Figure 14B, of the main effects (widening and lengthening) of the Helmholtz's Square as in Figure 14C are perceived.

These results of the phenomenological and scaling tasks are corroborated by those of the adjustment/matching task. First of all, the outcomes of the adjustment/matching task confirm previous results on the classical Helmholtz's Squares. The whole bunch of vertical stripes (left side of Figure 14C) was perceived to be of equal height with a pattern of horizontal stripes when it is between 4% and 10% taller. A paired sample *t*-test revealed a significant difference between the mean PSEs (*t* = 2.7, *df* = 12, *p* < 0.05). Therefore, when both bunches are the same height, the horizontal segments were perceived as being taller. Similarly, the set of horizontal stripes was perceived to be of equal width as the set of vertical stripes when it is between 2 and 8% wider. Namely, when both sets are the same width, the verticals were perceived as being wider.

Moreover, the results of all the paired comparisons among the stimuli were significantly different. More specifically for our purposes, when the two sources of directional organization are pitted one in favor of the other (Figure 14A), the whole square shapes yielded a significantly greater size illusion than did the classical versions of Figure 14C (left: *t* = 3.1, *df* = 12, *p* < 0.05; right: *t* = 3.5, *df* = 12, *p* < 0.05). Finally, accordingly to our hypotheses, when one source of directional organization is pitted against the other (Figure 14B), the size effects are reduced (left: *t* = 3.5, *df* = 12, *p* < 0.05; right: *t* = 2.8, *df* = 12, *p* < 0.05).

**Figure 15:**
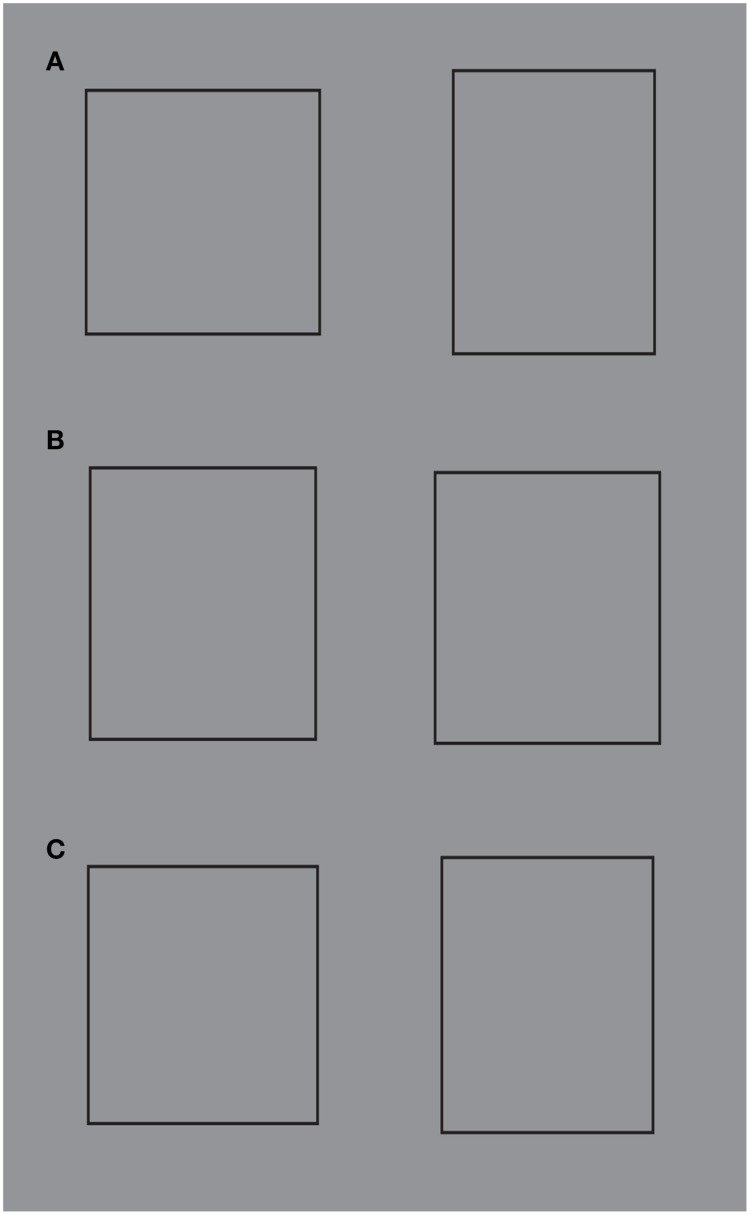
**The average squares matched with the conditions illustrated in Figure 14**. For further details please see the text.

Figure 15 illustrates the average squares matched with the stimulus conditions illustrated in Figure 14. The average sizes obtained were respectively: 30.865 × 27.019 (W × H, Figure 15A-left), 26.6 × 31.4 (Figure 15A-right), 29.8 × 30.109 (Figure 15B-left), 29.687 × 30.03 (Figure 15B-right), 30.3 × 28.5 (Figure 15C-left), 27.9 × 30.5 (Figure 15C-right).

**Figure 16:**
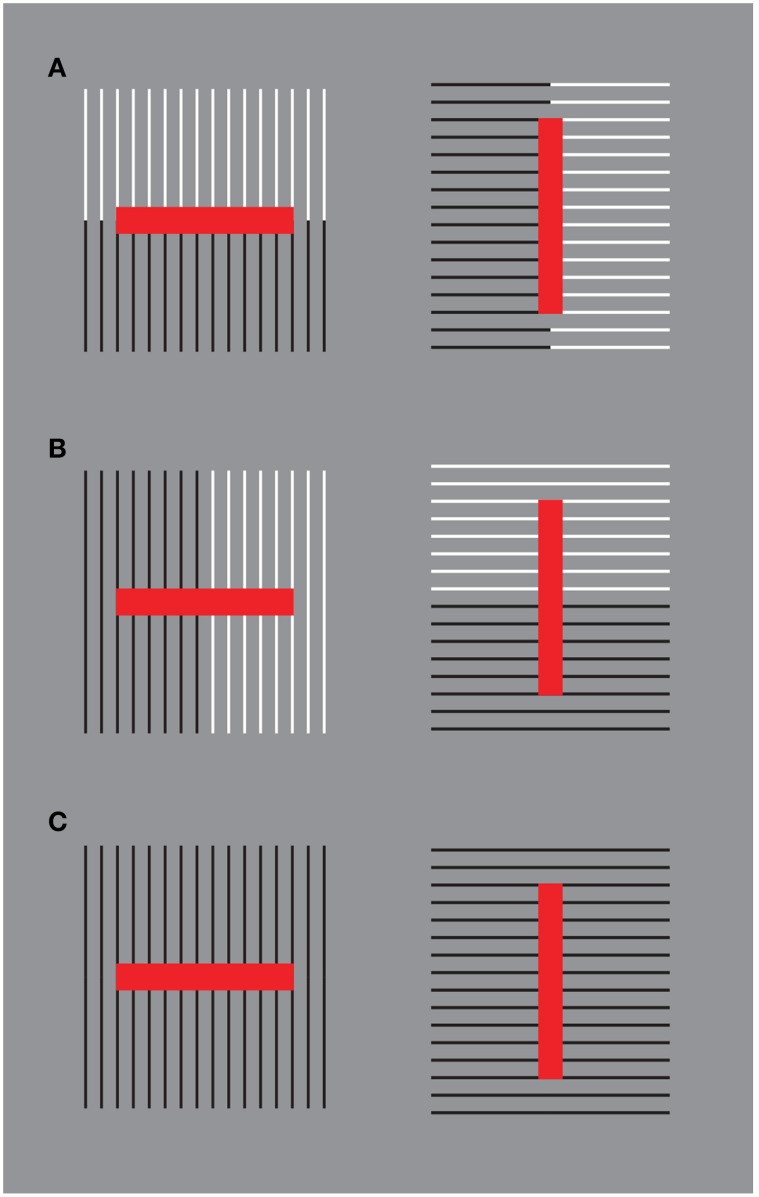
**By introducing other sources of directional organization the size effects can be further enhanced or weakened**. For further details please see the text.

The results of the phenomenological and psychophysical methods suggest that the previous effects can be further enhanced or weakened by introducing other sources of directional organization within the bunch of lines of Figure 14. A possible way among many is shown and demonstrated in Figure 16.

## Discussion and conclusions

On the base of the results shown in this and in the previous sections, starting from the insights of Gestalt psychologists at least three main points can be fixed. First of all, the problem of shape formation can be reconsidered in terms directional organization as resultant of the interaction between different sources. Second, these different sources can compete and cooperate. Third, phenomena apparently antinomic like the rectangle illusion and Helmholtz's squares can be resolved.

All these effects demonstrate that the grouping influences the shape. Under our conditions, the directional organization emerges from the Gestalt grouping principles, but it goes beyond them by assigning shape attributes. This is something new that goes beyond the Gestalt theory of grouping.

As a matter of fact, according to Gestalt psychologists, the grouping principles define the rules of “what is it or what stays with what” and, therefore, the grouping, not the shape. More precisely, they cannot describe and explain squares that appear like a vertical/horizontal rectangles or like diamonds and *vice versa*. Conversely, the notion of directional organization cannot be assimilated to or considered like a grouping principle. In fact, it does not define the grouping but the shape.

Moreover, while all the Gestalt grouping principles are placed at the same phenomenal plane, so that they can compete or support synergistically each other, the grouping principles and the directional shape organization are placed at different perceptual levels. As previously suggested, the directional organization behaves like some kind of meta-principle that operates at another organization level after and on the basis of the results of the perceptual grouping. In fact, as already mentioned, this distinction between principles and meta-principles or between grouping principles and shape principles is not trivial or merely linguistic but mostly phenomenological as demonstrated in the previous section. On the basis of these arguments and of the previous phenomenal results, we support the necessity to introduce the notion of “directional organization” as a complementary development of the grouping.

In conclusion, the new phenomena illustrated in this work demonstrates that the problem of shape formation can be profitably reconsidered in the light of the notion of directional organization with vectorial properties defined phenomenally as the visual property of being directional, accentuating, maintaining and polarizing a direction and coming from different geometrical sources. Consequently, the same figure can manifest different kinds of directional organization not necessarily synergistic, from whose complexity of interactions emerges a shape, with a rich set of attributes. The directional sources can compete and cooperate and, above all, they are all present at the same time, placed in a dynamic state of equilibrium that can be changed by accentuating another or the opposite competing source.

These phenomenological results can have interesting consequences not only by allowing the prediction of new phenomena but also in terms of neural circuitry.

### Conflict of interest statement

The author declares that the research was conducted in the absence of any commercial or financial relationships that could be construed as a potential conflict of interest.

## Supplementary material

Movie 1 **The perceived rotating shape switches from a diamond to a rotated square**.

Movie 2 **The perceived shape switches from a diamond to a rotated square**.
